# Effects of Resveratrol on Thymic Stromal Lymphopoietin Expression in Mast Cells

**DOI:** 10.3390/medicina57010021

**Published:** 2020-12-28

**Authors:** Phil-Dong Moon, Na-Ra Han, Jin Soo Lee, Hyun-Woo Jee, Ji-Hyeon Kim, Hyung-Min Kim, Hyun-Ja Jeong

**Affiliations:** 1 Center for Converging Humanities, Kyung Hee University, Seoul 02447, Korea; pdmoon@khu.ac.kr; 2 Department of Pharmacology, College of Korean Medicine, Kyung Hee University, Seoul 02447, Korea; nrhan@khu.ac.kr (N.-R.H.); mcjin21@naver.com (J.S.L.); 3 Department of Science in Korean Medicine, Graduate School, Kyung Hee University, Seoul 02447, Korea; aidenjee@naver.com (H.-W.J.); sjsdlsrk@naver.com (J.-H.K.); 4 Department of Food Science & Technology and Research Institute for Basic Science, Hoseo University, Chungnam 31499, Korea

**Keywords:** thymic stromal lymphopoietin, resveratrol, receptor-interacting protein2, caspase-1, intracellular calcium

## Abstract

*Background and objectives:* Cytokine thymic stromal lymphopoietin (TSLP) plays a pivotal role in the pathogenesis of atopic diseases such as atopic dermatitis, allergic rhinitis, and asthma. Resveratrol (RSV) exerts various pharmacological effects such as antioxidant, anti-inflammatory, neuroprotective, and anticancer. Although, it has been verified the beneficial effects of RSV on various subjects, the effect of RSV on thymic stromal lymphopoietin (TSLP) regulation has not been elucidated. *Materials and Methods:* Here, we examined how RSV regulates TSLP in HMC-1 cells. Enzyme-linked immunosorbent assay, real-time polymerase chain reaction, Western blotting, and calcium assay were performed to evaluate the effect of RSV. *Results:* TSLP production and mRNA expression were reduced by RSV. RSV down-regulated nuclear factor-κB activation, IκBα phosphorylation as well as activation of receptor-interacting protein2 and caspase-1 in HMC-1 cells. In addition, RSV treatment decreased the up-regulation of intracellular calcium in HMC-1 cells. *Conclusions:* These results suggest that RSV might be useful for the treatment of atopic diseases through blocking of TSLP.

## 1. Introduction

Atopic dermatitis (AD) is a common chronic skin disease worldwide [1,2,3]. The prevalence of AD is 2–10% of adults and 5–20% of children throughout the world [4]. The lifetime prevalence of AD has elevated over the past decades, particularly in industrialized countries [5]. AD induced a poor quality of life. Want of sleep, school or work absenteeism, and psychological stress arose from AD. Medical costs were elevated compared with those of patients without AD [6].

Cytokine thymic stromal lymphopoietin (TSLP) plays a pivotal role in the pathogenesis of atopic diseases such as atopic dermatitis, allergic rhinitis, and asthma. Intradermal injection of recombinant TSLP up-regulated scratching frequency in a murine AD model [7]. TSLP knockout mice showed decreased AD-like skin lesions [8]. Staphylococcal enterotoxin B plus 2,4-dinitrochlorobenzene induced significant TSLP elevation in AD-like skin lesions [9]. Epithelial cells and keratinocytes play important roles in AD; however, mast cells also contribute to the induction of AD [10]. Numerous reports suggest that mast cell population and mast cell activation were elevated in AD, indicating the significance of mast cells in AD [11,12,13].

Caspase-1 plays a role in inflammatory responses, though most caspases are involved in programmed cell death [14,15]. Ablation of caspase-1 decreased chemical-induced intestinal inflammation [16]. When cells are exposed to pro-inflammatory stimuli, receptor-interacting protein (RIP)2 binds, and activates caspase-1 [17]. Caspase-1/nuclear factor (NF)-κB signaling controlled TSLP expression [18]. Additionally, NF-κB activation decreased by caspase-1 inhibitor treatment, presenting that NF-κB is a downstream factor of caspase-1 [18].

**Figure 1:**
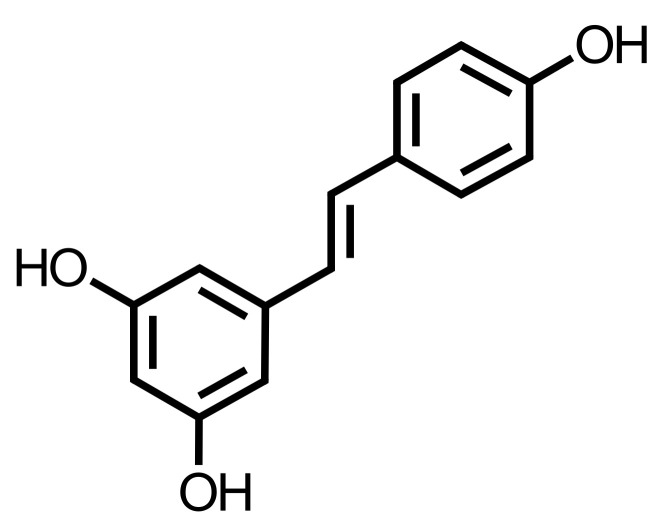
Chemical structure of RSV.

Resveratrol (RSV, Figure 1) is a dietary polyphenol found in various plants such as grapes, pines, apples, knotweed, blueberries, plums, and peanuts [19,20]. RSV exerts various pharmacological effects such as antioxidant, anti-inflammatory, neuroprotective, and anticancer [21,22,23]. Recently, Cheng and colleagues [24] reported that RSV ameliorates renal damage in obese mice via protection of inflammation. However, the beneficial effects of RSV on TSLP expression in mast cells have not been fully understood. In the present study, we studied whether RSV can regulate the TSLP expression in HMC-1 human mast cell line.

## 2. Materials and Methods

### 2.1. Reagents

We purchased RSV, phorbol myristate acetate (PMA), 3-[4,5-dimetylthiazol-2-yl]-2,5-diphenyltetrazolium bromide (MTT), dimethyl sulfoxide, calcium ionophore, and fetal bovine serum (FBS), from Sigma-Aldrich Corp. (St. Louis, MO, USA); TMB substrate from (Pharmingen, San Diego, CA, USA); Power SYBR^®^ Green PCR master mix from Applied Biosystems (Warrington, UK); penicillin/streptomycin mixture from Gibco BRL (Grand Island, NY, USA); TSLP antibodies from R&D Systems (Minneapolis, MN, USA); RIP2, caspase-1, GAPDH, phosphorylated (p)IκBα, and NF-κB p65 antibodies from Santa Cruz Biotechnology (Santa Cruz, CA, USA). 

### 2.2. Cells

HMC-1 cells were obtained from Dr. Yukihiko and maintained in IMDM with heat-inactivated FBS (10%), penicillin (100 U/mL), and streptomycin (100 μg/mL) at 37 °C in 5% CO2.

### 2.3. MTT Assay

MTT assay was used to analyze cytotoxicity as described previously [25]. Various concentrations of RSV (0.03, 0.3, and 3 μM) or PBS were pretreated in HMC-1 cells (3 × 10^5^/mL) for 1 h and incubated with 5 mg/mL of MTT for 4 h. MTT formazan was dissolved by 1 mL of dimethyl sulfoxide and transferred 200 μL of supernatant into a 96-well microplate. The plate was measured at 540 nm.

### 2.4. Enzyme-Linked Immunosorbent Assay (ELISA) 

Various concentrations of RSV (0.03, 0.3, and 3 μM) or PBS were pretreated in HMC-1 cells (3 × 10^5^/mL) for 1 h and incubated with PMA plus calcium ionophore (PMACI) for 7 h considering our previous reports [18,26]. An ELISA was utilized for assessment of TSLP levels from culture supernatants as described previously [27]. In short, anti-TSLP capture antibody was incubated on 96-well microplates overnight. After washing, TSLP standard solution and culture supernatants were added. After washing, anti-TSLP detection antibody was added. After washing again, avidin-peroxidase was incubated at 37 °C. Finally, substrate solution was added and the microplates were analyzed at 450 nm.

### 2.5. Real-Time Polymerase Chain Reaction (PCR)

Various concentrations of RSV (0.03, 0.3, and 3 μM) or PBS were pretreated in HMC-1 cells (1 × 10^6^/mL) for 1 h and incubated with PMACI for 5 h considering our previous reports [18]. Real-time PCR was performed by using a Power SYBR Green PCR master mix. ABI StepOne real-time PCR system (Applied Biosystems, Foster City, CA, USA) was used as described previously [28].

### 2.6. Extraction of Nuclear and Cytoplasmic Proteins

HMC-1 cells (5 × 10^6^/mL) were pretreated with RSV for 1 h before PMACI stimulation. Proteins were extracted by using hypotonic buffer and cold saline buffer as described previously [29,30].

### 2.7. Western Blotting

After incubation at 95 °C for 5 min, samples were cooled on ice. Each protein was separated by 15% SDS-polyacrylamide gel and transferred to nitrocellulose paper. Primary antibodies were treated overnight and then secondary antibodies were treated for 1 h. Protein bands were visualized using enhanced chemiluminescent reagent (Amersham Co., Newark, NJ, USA) as described previously [31].

### 2.8. Calcium Assay

For fluorescence measurement, fura-2/AM was added in HMC-1 cells (1 × 10^5^/mL) for 30 min. The cells then were harvested to perform the calcium assay as described previously [32].

### 2.9. Statistics

All results were analyzed by IBM SPSS software (version 23, SPSS Inc., Chicago, IL, USA). Statistical analysis was conducted by using independent t-test and ANOVA with a Tukey post hoc test. The results were considered significant at values of *p* < 0.05 and expressed as the mean ± standard error of the mean (SEM).

## 3. Results

### 3.1. Effect of RSV on TSLP Production

**Figure 2:**
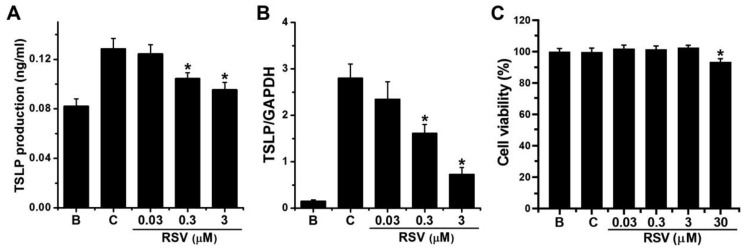
Effects of RSV on the production and mRNA expression of TSLP. (**A**) TSLP production was analyzed with ELISA. (**B**) TSLP mRNA expression was analyzed with real-time PCR. (**C**) Cell viability was analyzed with MTT assay. (**B**), PBS-added group; (**C**), PBS plus PMACI-added group. * indicates *p* < 0.05 versus the control (PBS plus PMACI) group.

PMA plus calcium ionophore (PMACI) were used to induce mast cell activation. To determine if RSV influences TSLP production in mast cells, HMC-1 cells were seeded in 24-well plates and pretreated with different doses of RSV (0.03, 0.3, and 3 μM) for 1 h. PMACI were able to induce TSLP production, which could be reduced by RSV (0.3 and 3 μM, Figure 2A). Incubation with PMACI (0.128 ± 0.008 ng/mL) increased TSLP production as compared to incubation with PBS (0.082 ± 0.006 ng/mL). In treatment with 0.03, 0.3, and 3 μM of RSV for 8 h, TSLP production was shown as 0.124 ± 0.008 ng/mL, 0.104 ± 0.005 ng/mL, and 0.095 ± 0.006 ng/mL, respectively. RSV alone did not show any TSLP increment compared with that in PBS-treated group (data not shown). Various concentrations of RSV (0.03, 0.3, 3, and 30 μM) were pretreated in HMC-1 cells considering the report of Bollmann et al. [33]; however, 30 μM of RSV showed cytotoxicity (Figure 2C). Thus, we selected 0.03, 0.3, and 3 μM.

### 3.2. Effect of RSV on TSLP mRNA Expression

To investigate if RSV influences TSLP mRNA expression in mast cells, HMC-1 cells were seeded in 6-well plates and pretreated with various doses of RSV (0.03, 0.3, and 3 μM) for 1 h. PMACI were able to induce TSLP mRNA expression, which could be reduced by RSV (0.3 and 3 μM, Figure 2B). Incubation with PMACI (2.797 ± 0.307) increased TSLP mRNA expression as compared to incubation with PBS (0.150 ± 0.029). In treatment with 0.03, 0.3, and 3 μM of RSV for 6 h, TSLP mRNA expression was shown as 2.347 ± 0.377, 1.610 ± 0.195, and 0.727 ± 0.149, respectively. Because the efficacy at 3 μM RSV was better than 0.03 and 0.3 μM, we evaluated the effect of RSV at 3 μM on following experiments (NF-κB activation, IκBα phosphorylation, RIP2 activation, caspase-1 activation, and intracellular calcium level).

### 3.3. Effects of RSV on Activation of NF-κB and Phosphorylation of IκBα

**Figure 3:**
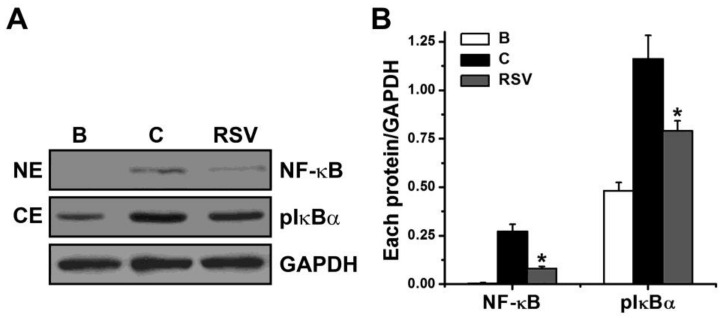
Effects of RSV on the activation of NF-κB and phosphorylation of IκBα. (**A**) Each protein was analyzed with Western blot analysis. Representative immunoblots are shown. (**B**) Each band was quantitated by densitometry. NE, nuclear extract; CE, cytoplasmic extract; B, PBS-added group; C, PBS plus PMACI-added group. * indicates *p* < 0.05 versus the control (PBS plus PMACI) group.

To evaluate if RSV influences NF-κB and IκBα signal cascade in mast cells, HMC-1 cells were seeded in 6-well plates and pretreated with RSV (3 μM) for 1 h. PMACI were able to induce NF-κB p65 activation, which could be reduced by RSV (3 μM, Figure 3). Incubation with PMACI (0.271 ± 0.038) increased NF-κB activation as compared to incubation with PBS (0.004 ± 0.003). In treatment with 3 μM of RSV for 2 h, NF-κB activation was shown as 0.081 ± 0.011. NF-κB activation resulted from IκBα phosphorylation [34]. Thus, we examined if RSV influences the IκBα phosphorylation. PMACI were able to induce IκBα phosphorylation, which could be reduced by RSV (3 μM, Figure 3). Incubation with PMACI (1.162 ± 0.122) increased IκBα phosphorylation as compared to incubation with PBS (0.482 ± 0.042). In treatment with 3 μM of RSV for 2 h, IκBα phosphorylation was shown as 0.792 ± 0.051.

### 3.4. Effects of RSV on Activation of RIP2 and Caspase-1

**Figure 4:**
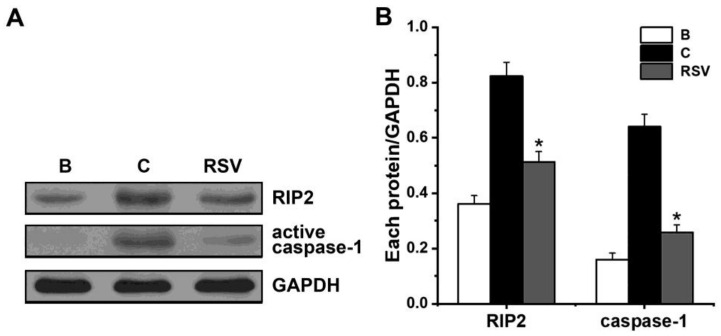
Effects of RSV on the activation of RIP2 and caspase-1. (**A**) Each protein was analyzed with Western blot analysis. Representative immunoblots are shown. (**B**) Each band was quantitated by densitometry. B, PBS-added group; C, PBS plus PMACI-added group. * indicates *p* < 0.05 versus the control (PBS plus PMACI) group.

To examine if RSV influences the activation of RIP2 and caspase-1 in mast cells, we seeded HMC-1 cells in 6-well plates and pretreated them with RSV (3 μM) for 1 h. PMACI were able to induce activation of RIP2 and caspase-1, which could be reduced by RSV (3 μM, Figure 4). Incubation with PMACI (0.823 ± 0.051 for RIP2; 0.640 ± 0.045 for caspase-1) increased caspase-1 activation as compared to incubation with PBS (0.361 ± 0.030 for RIP2; 0.161 ± 0.023 for caspase-1). In treatment with 3 μM of RSV for 1 h, levels for activation of RIP2 and caspase-1 were shown as 0.514 ± 0.037 and 0.258 ± 0.027, respectively.

### 3.5. Effect of RSV on Calcium Levels

**Figure 5:**
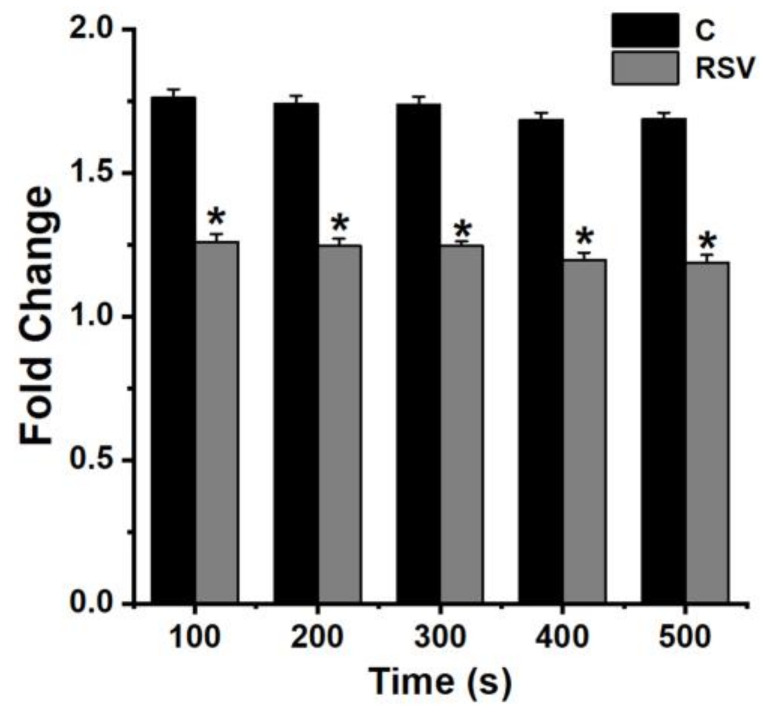
Effect of RSV on intracellular calcium. Calcium levels were analyzed using Fura-2/AM for 500 s with a spectrofluorometer (excitation 360nm, emission 450nm, Thermo Fisher Scientific Inc.). C, PBS plus PMACI-added group. * indicates *p* < 0.05 versus the control (PBS plus PMACI) group.

Calcium is essential for the activation of RIP2 and caspase-1 [35,36]. To determine if RSV influences the intracellular calcium in mast cells, HMC-1 cells were seeded in 96-well plates and pretreated with RSV (3 μM) for 20 min. PMACI were able to induce elevation of intracellular calcium, which could be reduced by RSV (3 μM, Figure 5).

## 4. Discussion

In this study, RSV reduced the TSLP production and mRNA expression. Additionally, RSV attenuated the NF-κB activation, IκBα phosphorylation as well as RIP2/caspase-1 activation. Lastly, RSV inhibited intracellular calcium levels.

In general, mast cell activation results from the binding of IgE receptors with multivalent antigens. Activation of protein kinase C (PKC), as well as increment of intracellular calcium are shown in activated mast cells [37]. To make a similar condition, PMA was used to activate PKC, and calcium ionophore was used to increase intracellular calcium in this experiment. TSLP production and mRNA expression were elevated by incubation with PMACI in HMC-1 cells [18]. Serum TSLP levels increased in patients with AD compared with those of healthy subjects [38]. Diesel exhaust particles increased mRNA and production of TSLP from bronchial asthmatic epithelial cells that isolated from bronchus of patients with asthma [39]. The results of this study also presented that the production and mRNA expression of TSLP are reduced by RSV treatment in HMC-1 cells (Figure 2). It will be possible to suggest that RSV may contribute to the treatment for atopic and asthmatic diseases.

A study suggested that TSLP was regulated by NF-κB in epithelial cells [40]. Additionally, TSLP production and mRNA expression were regulated by NF-κB in mast cells [18]. Moreover, it has been suggested that NF-κB plays a critical role in the production of TSLP [41]. Our results presented that NF-κB activation and IκBα phosphorylation were down-regulated by RSV (Figure 3), suggesting that inhibition of TSLP by RSV is regulated by NF-κB/IκBα in HMC-1 cells. External treatment of NF-κB inhibitor (IMD-0354) reduced AD symptoms [42]. Jiang et al. [43] suggested that topical treatment of specific NF-κB inhibitor (dehydroxymethylepoxyquinomicin) improves 2,4-dinitrochlorobenzene and oxazolone-induced AD-like skin lesions. In recent research, AD symptoms were ameliorated by topical treatment of specific NF-κB inhibitor (dehydroxymethylepoxyquinomicin) in chemical irritants plus horny layer removing-induced AD murine model [44]. Thus, we presume that RSV may be helpful for us to improve AD symptoms via blocking of NF-κB.

When cells are exposed to pro-inflammatory stimuli, RIP2 binds and activates caspase-1 [17]. Numerous studies demonstrated that activation of RIP2 and caspase-1 resulted from pro-inflammatory stimuli including PMACI or ovalbumin [14,45,46]. Our findings presented that the activation of RIP2 and caspase-1 was reduced by RSV (Figure 4). The results of the present study implied that RSV might be active in reducing TSLP through blocking RIP2 and caspase-1 in HMC-1 cells.

**Figure 6:**
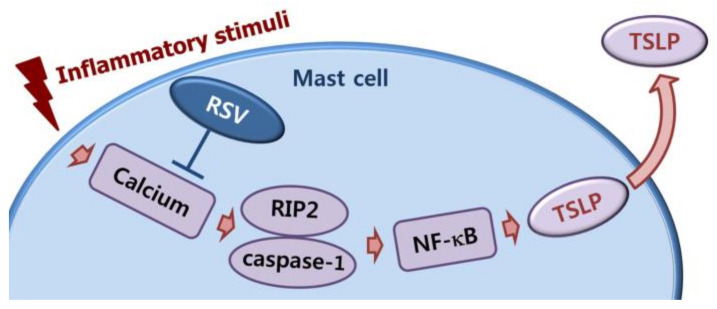
A schematic diagram of TSLP reduction by RSV. When mast cells are exposed to pro-inflammatory stimuli, intracellular calcium levels were elevated, the elevated intracellular calcium resulted in activation of RIP2 and caspase-1. NF-κB activation resulted from the activation of RIP2 and caspase-1. Lastly, the NF-κB activation resulted in TSLP production. In the present study, RSV decreased the TSLP production through blocking of calcium/RIP2/caspase-1/NF-κB signals in mast cells.

Increment of intracellular calcium induced activation of RIP2 and caspase-1 [35,36]. Han et al. [35] reported that calcium chelator BAPTA-AM decreases the protein levels of RIP2 and caspase-1. Our results presented that intracellular calcium was reduced by RSV (Figure 5). Hence, the results implied that RSV may reduce TSLP through down-regulating calcium/RIP2/caspase-1/NF-κB signals in HMC-1 cells (Figure 6).

Finally, daily administration of RSV (300 mg/kg) for 4 weeks did not show any toxicity in rats [47]. In the present study, we treated 3 μM of RSV (approximately 0.684 mg/kg). Thus, we presuppose that 3 μM of RSV would not be toxic to humans.

## 5. Conclusions

Collectively, the present study showed that RSV reduced TSLP production and mRNA expression. RSV inhibited NF-κB activation, IκBα phosphorylation, as well as activation of RIP2 and caspase-1 in HMC-1 cells. Furthermore, RSV reduced intracellular calcium levels. Therefore, these findings suggest that RSV might have important implications for screening promising drugs in atopic diseases.

## Data Availability

The data of this study are available from the corresponding author upon reasonable request.
